# Thyroid Storm: Unusual Presentation and Complication

**DOI:** 10.7759/cureus.12483

**Published:** 2021-01-04

**Authors:** Maan A Albehair, Abdulrahman A Alagga, Weaam Z Ghulam, Abdullatif M Alomair, Dunya AlFaraj

**Affiliations:** 1 College of Medicine, Imam Abdulrahman Bin Faisal University, Dammam, SAU; 2 Emergency, King Fahad University Hospital, Khobar, SAU; 3 Emergency Medicine, Imam Abdulrahman Bin Faisal University, Dammam, SAU

**Keywords:** epstein-barr virus (ebv), fulminant hepatic failure, congestive heart failure, thyroid storm

## Abstract

Thyroid storm is a rare and a life-threatening condition, and serious complications could happen if not diagnosed and managed early. The typical clinical manifestations of hyperthyroidism are exaggerated in thyroid storm, particularly marked pyrexia and tachycardia, and altered mental status as agitation, delirium, or coma. Many precipitating factors contribute to the presentation of thyroid storm, and new recent factors like Epstein-Barr virus (EBV) could play a significant role. Serious and rare complications from the thyroid storm can increase the risk of mortality and morbidity up to 30% as fulminant hepatic failure, which is reported only a handful of times in the literature. Also, congestive heart failure can be part of the multiorgan dysfunction from the presentation, if associated with the thyroid storm.

In this report, we present a case of thyroid storm precipitated by EBV and causing fast atrial fibrillation complicated by congestive heart failure and fulminant hepatic failure for 46-year-old Bangladeshi male not known to have any medical illness. He presented to the emergency department with 10-day history of an on and off subjective fever, associated with generalized abdominal pain and vomiting. He developed palpitation at the day of presentation. He was managed, stabilized, intubated, and shifted to the ICU as the patient started to be apneic after the conscious sedation for the cardioversion. During the admission anti-EBV viral capsid antigen IgM antibody was positive. The patient was discharged after five days in ICU and 14 days of overall hospital stay.

Although the complication of thyroid storm as fulminant hepatic failure and congestive heart failure are rare, they should be considered in cases with thyroid storm. The pre-existing of EBV as a precipitating factor should always be considered, and more studies in these regards need to be done.

## Introduction

Thyroid emergencies are not common; hence they are very serious life-threatening conditions. Thyroid storm and myxedema coma were described a long time ago, and many types of research were conducted to reach the pathophysiology and the most accurate way to manage such cases. The seriousness of these conditions is behind being undiagnosed or untreated thyroid disease, respectively, hence raising the possibility of having a serious, unpredicted clinical deterioration. More often, it is an acute reaction to thyroid or nonthyroidal surgery, trauma, infection, or stress-associated event. In 1926, the first thyroid storm was described as ‘‘the crisis of exophthalmic goiter’’ by Lahey.

The typical clinical manifestations of hyperthyroidism are exaggerated in thyroid storm, particularly marked pyrexia, tachycardia, and altered mental status as agitation, delirium, or coma, which are common features. These findings, coupled with the clinical picture of a patient with hyperthyroidism, lid lag, stare, goiter, ophthalmopathy, and tremor, should alert the clinician to the diagnosis. The diagnosis is mainly made based on clinical criteria as thyroid hormone measurements do not help in differentiating between thyroid storm and hyperthyroidism. Survival of thyroid storm can only be optimized by the early diagnosis as we mentioned based on clinical criteria and prompt initiation of multimodal therapy, including inhibition of thyroid hormone synthesis and secretion as well as inhibition of thyroid hormone effects in the periphery and supportive measures. Overall, 10% to 30% mortality remains high even with early diagnosis.

Furthermore, there are rare and severe presentations for the thyroid storm, like what we faced in our case. Few reported cases of the uncommon complication of thyroid storm, such as Fulminant hepatic failure, will increase the risk of mortality and permanent complication if associated or secondary to congestive heart failure. It has been noticed that atrial fibrillation is the most common cardiac complication of hyperthyroidism and occurs in 15% of patients with hyperthyroidism, as it is associated with a higher risk of thromboembolism that often involves the central nervous system. It has recently been reported that Epstein-Barr virus is considered a contributing factor of thyroid storm [1-3]. We will present a rare case of thyroid storm, which is precipitated by Epstein-Barr virus (EBV) and causing fast atrial fibrillation complicated by congestive heart failure and fulminant hepatic failure.

## Case presentation

A 46-year-old Bangladeshi male, not known to have any medical or surgical illness, not on any medication, presented to the emergency department with 10-day history of subjective fever on and off, associated with generalized abdominal pain and vomiting multiple times, and he developed palpitation on the day of presentation. He denied any history of altered mental status, loss of consciousness, syncope, chest pain, diarrhea, or constipation.

On physical examination his heart rate (HR) was 140-150 beats per minute, respiratory rate (RR) was 27 breaths per minute, and other vitals were within the normal range. The patient looked ill and on pain distress. He was conscious, alert, oriented to time, place, and person. Brisk carotid pulse and distended neck veins were noticed. Chest was unremarkable. Abdomen was distended, soft, and lax. He had right upper quadrant mild tenderness with hepatomegaly. Regarding face examination, the patient had exophthalmos; otherwise, normal facial examination.

**Figure 1:**
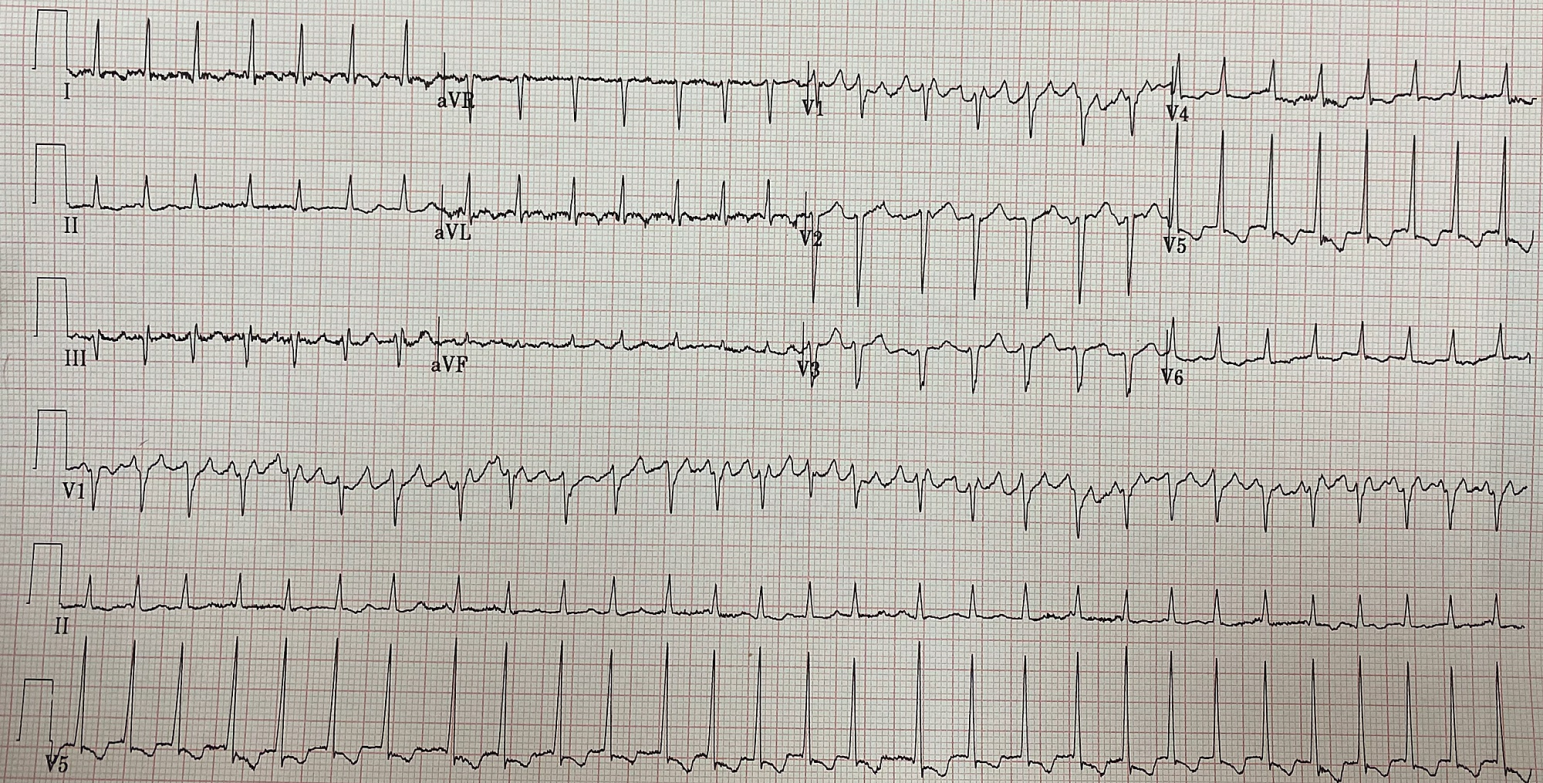
Electrocardiogram (ECG) of the patient before the cardioversion

The patient's 12-lead electrocardiogram (ECG) was done, which showed atrial fibrillation with rapid ventricular response (Figure 1). His bedside echocardiogram showed severe left ventricular ejection fraction (LVEF) of 30%, global hypokinesis, impaired right ventricular function, dilated atria, dilated inferior vena cava, and high filling pressure. Significant laboratory findings were: highly elevated liver enzymes, very low thyroid-stimulating hormone (TSH), high T3, and T4, lactic acid is elevated, prolonged PT, and abnormal international normalized ratio (INR), as shown in Table 1.

**Table 1:** Laboratory findings at the time of admission LFT: Liver function test; AST: Aspartate aminotransferase; ALT: Alanine aminotransferase; LDH: Lactate dehydrogenase; INR: International normalized ratio; aPTT: activated partial thromboplastin time; TSH: Thyroid stimulating hormone; CRP: C-reactive protein; PTH: Parathyroid hormone; PCR: Polymerase chain reaction; PT: Prothrombin time.

Labs	Value	Normal range
LFT		
Total bilirubin	8.3 mg/dL	0.2-1.2 mg/dL
Direct bilirubin	4.7 mg/dL	0.1-0.5 mg/dL
Total protein	6.7 g/dL	6.4-8.3 g/dL
Albumin	3.0 g/dL	3.2-5.2 g/dL
Alkaline Phosphatase	283 U/L	40-150 U/L
SGOT (AST)	1306 U/L	5-34 U/L
SGPT (ALT)	921 U/L	7-55 U/L
LDH	1286 U/L	81-234 U/L
Coagulation profile		
PT	31.3 sec	12.9-15.9 sec
INR	2.83	
aPTT	47.6 sec	25.6-42.3 sec
Thyroid hormones		
TSH	<0.0083 uLU/mL	0.35-4.94 uLU/mL
Free T3	5.59 pg/ml	1.88-3.18 pg/ml
Free Thyroxine	2.30 ng/dL	0.70-1.48 ng/dL
Other labs		
Lactic acid	6.57 mmol/L	0.5-2.2 mmol/L
SARS-Cov-2 PCR	Negative	
Procalcitonin	0.27	0.1 or less
CRP	5.93 mg/dL	0.10-0.5 mg/dL
Intact PTH	53.59 pmol/L	1.58-7.2 pmol/L

The first differential diagnosis that came to our mind was thyroid storm, and the decision was made to treat accordingly.

Since the patient had the picture of heart failure, and beta-blocker is controversial to use. So, a small dose of propranolol was given (1 mg). The patient’s condition started to deteriorate within two minutes after propranolol administration (he received half of the dose by that time). He started to become disoriented and sweaty, so the rest of the dose was held.

Shortly after stopping propranolol, he returned to normal mental status, but he was still tachycardic, and his rhythm showed fast atrial fibrillation. Suddenly, he started to be hypoxic, so he was placed on bilevel positive airway pressure (BiPAP). But he did not tolerate it, so BiPAP was removed, and he was placed on a nonrebreather mask with good response. The patient had unstable atrial fibrillation, which was associated with his acute heart failure, which was most likely secondary to thyroid storm. Thus, the initial treatment with propranolol failed. A shared decision with the cardiologists was made to do DC (Direct Current) cardioversion. The patient was sedated with ketamine and propofol, and then a synchronized cardioversion 100 J was given. The patient returned to normal sinus rhythm but started to be apneic, so intubation was done, and the patient shifted to the ICU.

During the admission, multiple consultations for multiple services to confirm the diagnosis and more laboratory tests were done. Laboratory tests were as follows:

- Monospot test for infectious mononucleosis screen was negative.

- Anti-EBV viral capsid antigen IgM antibody was positive.

- All toxicology screenings were negative.

Thyroid storm was confirmed by BWPS (Burch-Wartofsky Point Scale) (Table 2), which was 65 points (25 as tachycardia more than 140, 10 points for the presence of atrial fibrillation, 20 as severe congestive heart failure, and 10 points as for vomiting and abdominal pain) [4].

**Table 2:** Burch-Wartofsky Point Scale (BWPS)

Criteria	Points
Thermoregulatory dysfunction	
Temperature (°C)	Points
37.2-37.7	5
37.8-38.3	10
38.4-38.8	15
38.9-39.4	20
39.5-39.9	25
More than 40.0	30
Cardiovascular	
Tachycardia (beats per minute)	Points
100-109	5
110-119	10
120-129	15
130-139	20
140 and above	25
Atrial fibrillation	Points
Absent	0
Present	10
Congestive heart failure	
Absent	0
Mild	5
Moderate	10
Severe	20
Gastrointestinal-hepatic dysfunction	
Absent	0
Moderate (diarrhea, abdominal pain, nausea/vomiting)	10
Severe (jaundice)	15
Central nervous system disturbance	
Absent	0
Mild (Agitation)	10
Moderate (delirium, psychosis, extreme lethargy)	20
Severe (seizure, coma)	30
Precipitating event	
Absent	0
Present	10
Interpretation	
Total score	Impression
45 and more	Thyroid storm
25-45	Impending storm
25 and less	Storm unlikely

**Figure 2:**
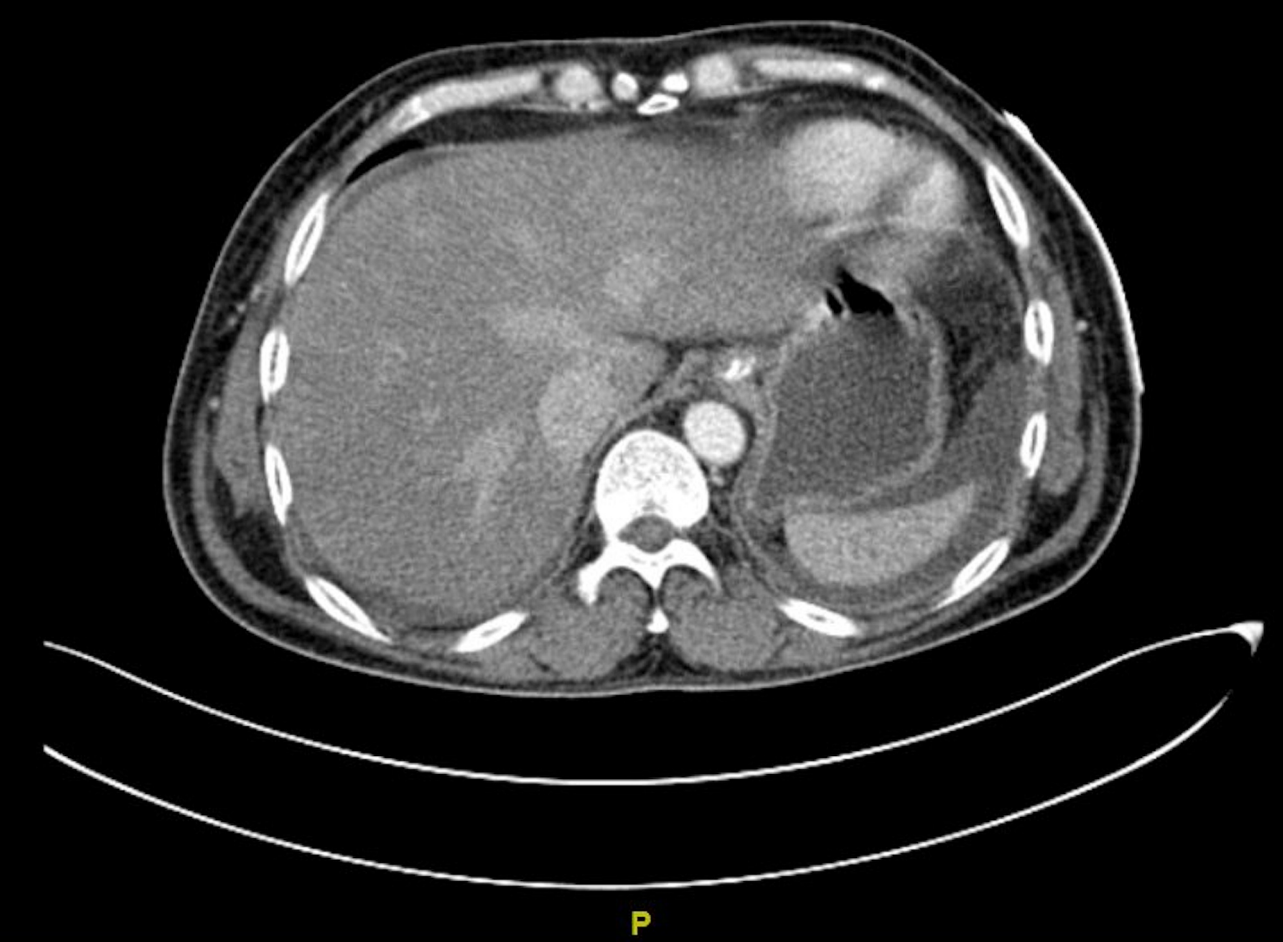
CT scan of the liver

Thyroid ultrasound was done which showed that both thyroid gland lobes were mildly enlarged in size, slightly wavy surfaces, and moderately heterogeneous parenchymal echotexture with moderately increased vascularity. Color Doppler scan gave the impression of diffuse goiter with signs of autoimmune thyroiditis (Figure 2).

**Figure 3:**
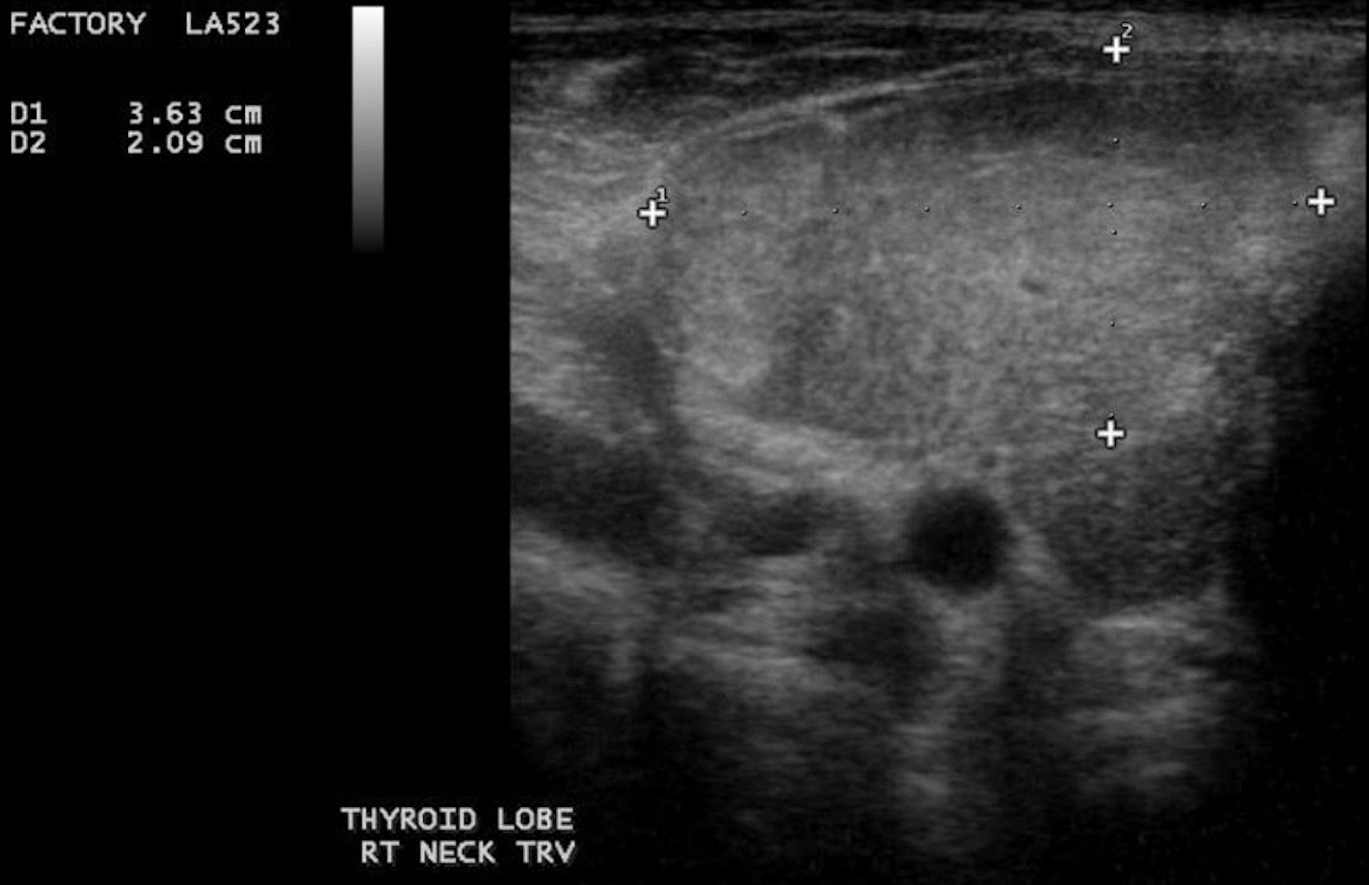
Ultrasound of the right thyroid lobe

Fulminant hepatic failure with liver congestion was seen by CT scan. CT scan showed a liver of normal size with mosaic hypo-enhancement and mottled appearance. There was a reflux of contrast within the dilated inferior vena cava and hepatic veins giving the impression of passive hepatic congestion (Figure 3).

After one-month follow-up, the patient's symptoms improved, and the last TSH level tested was within the normal range (3.0 uLU/mL).

## Discussion

Thyroid storm is a sudden life-threatening episode of thyrotoxicosis. Thyroid storms usually present in patients with a known hyperthyroidism disorder. Nevertheless, some studies reported variable presentations of atypical thyroid storm, including acute abdominal pain, seizures, heart failure, hepatic failure, and cerebral infarction [5].

Fulminant hepatic failure (FHF) is defined as encephalopathy with severe coagulopathy that occurs within eight weeks of the onset of symptoms of liver disease. Jaundice is usually the first symptom of liver failure, which begins the measured timeline to FHF. FHF is an extremely rare complication of thyroid storm as there are few reported cases of FHF associated with thyroid storm. In 2013, Hambleton et al. reported a case of thyroid storm and FHF along with a literature review of the evidence of thyroid storm and FHF, where they state that only seven similar cases have been reported. There is a 40% mortality rate for reported patients who were treated medically. From these seven cases, two of them died, and two cases which were managed surgically by thyroidectomy survived without complication [6].

Another recent case report in September 2020 was of a 22-year-old female with a history of Grave’s disease who presented with a one-week history of abdominal pain, nausea, and vomiting and diagnosed with thyroid storm with acute hepatic failure; she was managed medically with improvement after one-month follow-up [7].

In our case, the similarity with the few reported cases is present. However, the most surprising is that the patient was medically free before presenting to the emergency department with a thyroid storm complicated by FHF as the patient had had no medical history of either thyroid or liver disease before. He was managed medically with improvement and got discharged within one week of hospital admission.

Multiorgan dysfunction is one of the severe and life-threatening complications of the thyroid storm, which could lead to mortality if not managed at the time of presentation. Multiorgan dysfunction is a big concern to be considered in any patient coming with a thyroid storm.

The first case of thyroid storm to be reported with a presentation of multiorgan dysfunction was in 2000 [5]. It was a case of a 44-year-old female with a history of progressive dyspnea on exertion for two months associated with fatigue, hyperphagia, diarrhea, and weight loss. Investigations showed elevated liver function test (LFT) and renal function test (RFT) and congestive heart failure, ending up with thyroid storm as a diagnosis [5]. Another case was reported in 2010, of a 35-year-old woman who was a known case of Grave’s disease, incompliant to her thyroid medications. She presented to the ER with rapid atrial fibrillation and heart failure; investigations and clinical presentation confirmed thyroid storm diagnosis [8].

It has been controversial that using propranolol in case of heart failure is contraindicated due to its effect on the deterioration of the patient's hemodynamic status and leading to mortality. Furthermore, recent studies suggest the opposite, which is that propranolol is safe and well-tolerated and has beneficial effects on ventricular function in heart failure (HF) patients [9]. In our case, the patient deteriorated after receiving 1 mg of propranolol as he was having atrial fibrillation; the dose was stopped after the deterioration without completing it, which raises the susceptibility to propranolol to be the leading cause to the deterioration of the hemodynamic status of the patient as he was labeled HF.

Epstein-Barr virus (EBV) is the primary agent of infectious mononucleosis (IM), and it usually persists worldwide as subclinical infection. It is known that EBV is associated with lymphoproliferative disorders and malignancies in the long-term or chronic exposure [10]. Recent evidence supports the pre-existing EBV infection in thyroid cancer and thyroid tumor and it is found that EBV plays a significant role in developing thyroid tumor and thyroid cancer, especially papillary thyroid carcinoma in younger ages [11-13]. However, the presence of acute EBV infection does not usually cause thyroid storm or autoimmune thyroid disease, but recently EBV infection was reported in autoimmune thyroid disorders. Although EBV is not the only agent responsible for developing autoimmune thyroid diseases as it is not fully understood, it can be considered a contributory factor as in our case where the patient's thyroid ultrasound suggests the presence of autoimmune disease associated with acute EBV infection. However, the IgM test, where it was positive, could be lasting for three months, putting the possibility of linking EBV infection to hidden autoimmune thyroid disease leading to thyroid storm a strong possibility to be considered [3]. One study assumed that high prevalence of EBV infection in cases of Hashimoto's and Graves' diseases implies a potential etiological role of EBV in autoimmune thyroiditis. In some cases, it could represent a negative prognostic marker pointing to a higher risk of primary thyroid lymphoma development [14].

## Conclusions

At the end of this report, we can conclude that although Fulminant hepatic failure (FHF) is a rare complication of the thyroid storm, we should consider it more as it might lead to a higher rate of mortality if not managed early. Also, multiorgan dysfunction is seen in many patients as a complication of thyroid storm, which plays a significant role in the course of the clinical presentation and management of the case, which needs to be always investigated.

Recently the evidence supports the pre-existing Epstein-Barr virus (EBV) in thyroid storm cases, which may contribute to an unexpected clinical complication. Furthermore, studies are needed to address the relation between EBV and thyroid storm to enrich the current evidence along with the other complications.
